# Adjuvant Properties of Thermal Component of Hyperthermia Enhanced Transdermal Immunization: Effect on Dendritic Cells

**DOI:** 10.1371/journal.pone.0032067

**Published:** 2012-02-20

**Authors:** Neha Joshi, Vikas Duhan, Neelam Lingwal, Sangeeta Bhaskar, Pramod Upadhyay

**Affiliations:** National Institute of Immunology, Aruna Asaf Ali Marg, New Delhi, India; Oklahoma Medical Research Foundation, United States of America

## Abstract

Hyperthermia enhanced transdermal (HET) immunization is a novel needle free immunization strategy employing application of antigen along with mild local hyperthermia (42°C) to intact skin resulting in detectable antigen specific Ig in serum. In the present study, we investigated the adjuvant effect of thermal component of HET immunization in terms of maturation of dendritic cells and its implication on the quality of the immune outcome in terms of antibody production upon HET immunization with tetanus toxoid (TT). We have shown that *in vitro* hyperthermia exposure at 42°C for 30 minutes up regulates the surface expression of maturation markers on bone marrow derived DCs. This observation correlated *in vivo* with an increased and accelerated expression of maturation markers on DCs in the draining lymph node upon HET immunization in mice. This effect was found to be independent of the antigen delivered and depends only on the thermal component of HET immunization. *In vitro* hyperthermia also led to enhanced capacity to stimulate CD4+ T cells in allo MLR and promotes the secretion of IL-10 by BMDCs, suggesting a potential for Th2 skewing of T cell response. HET immunization also induced a systemic T cell response to TT, as suggested by proliferation of splenocytes from immunized animal upon *in vitro* stimulation by TT. Exposure to heat during primary immunization led to generation of mainly IgG class of antibodies upon boosting, similar to the use of conventional alum adjuvant, thus highlighting the adjuvant potential of heat during HET immunization. Lastly, we have shown that mice immunized by tetanus toxoid using HET route exhibited protection against challenge with a lethal dose of tetanus toxin. Thus, in addition to being a painless, needle free delivery system it also has an immune modulatory potential.

## Introduction

Needle-free devices for transdermal immunization can make vaccination painless, safe and affordable [1]. Several technologies including iontophoresis [2], sonophoresis [3], [4] microneedle delivery [5], [6] chemical permeation enhancement [7], [8] and particles or jet injection [9], [10] are being explored for this purpose. We have been investigating the hyperthermia enhanced transdermal (HET) immunization as a novel, without-needle immunization technology which is comparatively easy to generate and puts less strain on the recipient [11]. Febrile range mild hyperthermia is known to modulate the activation and maturation of dendritic cells both *in vitro* and *in vivo* [12]–[14] Use of hyperthermia in cancer vaccination has already been investigated and is being developed for stimulation of tumor-antigen bearing dendritic cells during adoptive transfer [15], [16].

The dendritic cells (DCs) are antigen presenting cells which are present in small number in tissues which are in contact with the external environment, mainly the skin, the inner lining of the nose, lungs, stomach, and intestine. They ingest antigens in peripheral tissues, migrate to lymph nodes and present MHCII bound antigen to CD4 T cells, which forms the basis of T cell immunity against bacterial antigens. The DCs are the most efficient antigen presenting cells as they have remarkable migratory capacity, optimal efficiency to capture and process antigens, and high level expression of MHC complex as well as co-stimulatory and adhesion molecules. The intricate pathways followed by DCs in these processes have been highlighted by some authors [17], [18].

Dermal dendritic cells and Langerhans cells are an important class of antigen presenting cells in the skin. The epidermal dendritic cells, upon encountering the antigen in periphery, are known to undergo maturation and migrate out of skin to the lymph node, where they present the antigens to T cells [19]. Various physical and chemical stimulants like LPS are known to cause the change in the co-stimulatory profile of DCs, thus affecting their antigen presenting capacity [20]. The cytokines secreted by DCs upon activation are known to influence the Th1/Th2 balance, in turn regulating the immune outcome in terms of the class of antibodies generated [21].

Here, we have investigated how the thermal component of this novel immunization technique influences the phenotype and functions of DCs *in vivo* and *in vitro*. We also examined the secretory profile of DCs upon HET immunization and how it skews the Th response, which in turn determines the antibody response generated *in vivo*. Also, we have shown that HET immunization generates protective immunity to toxin mediated paralysis and death. Thus, the current study establishes that besides being a non-invasive and painless method of immunization, HET immunization also have adjuvant properties, is able to skew the immune outcome in terms of antibody generation and T helper response, and provides protective immunity as well.

## Results

### *In vitro* hyperthermia up regulates the expression of activation markers on dendritic cells

**Figure 1:**
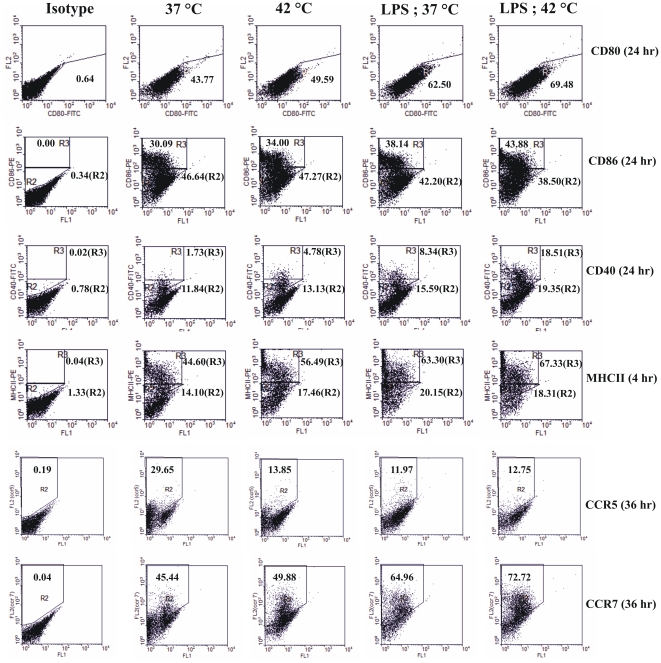
*In vitro* hyperthermia differentially regulates the expression of maturation markers on BMDCs. Immature or LPS (1 µg/ml; 6 hours) matured BMDCs were either maintained at 37°C or exposed to hyperthermia for 42°C for 30 minutes and analyzed by flow cytometry at indicated recovery periods, after staining with anti-CD80, anti-CD86, anti-MHC-II, anti-CD40, anti-CCR5 and anti-CCR7 mAbs. Results are shown as dot plots indicating the percentage of cells expressing the various markers and are representative of three independent experiments.

To study the effect of hyperthermia on activation of dendritic cells, BMDCs from day 7 of bone marrow culture were harvested and plated at a concentration of 1 million cells/ml in a 6- well plate. Immature as well as LPS (1 µg/ml; 6 hours) matured BMDCs were given *in vitro* hyperthermia (42°C for 30 minutes) followed by recovery at 37°C for indicated durations and the expression of activation markers CD80, CD86, MHCII and CD40 was assessed by flow cytometry. The recovery period was chosen on the basis of kinetics of expression of individual marker (data not shown). As shown in Figure 1, *in vitro* hyperthermia followed by recovery at 37°C caused up regulation of co-stimulatory molecules CD80 and CD86, antigen presentation marker MHCII as well as DC maturation marker CD40. Although the expression of CD80 was distinct, the expression of CD86, CD40, and MHCII in cultured cells was observed as two distinct populations, exhibiting that these markers are expressed at intermediate and high levels. The hyperthermia treatment further enhanced CD86-, CD40-, and MHCII-high expressing populations in immature and LPS matured cultures. Not only the *in vitro* hyperthermia caused an up regulation in maturation markers expression, it also enhanced the kinetics of maturation in terms of CD40 expression. The immature as well as LPS treated control groups show a peak of CD40 expression after 48 hours, whereas in the hyperthermia group, the expression peaked within 36 hours (Figure S2).

The process of antigen presentation by dendritic cells requires the migration of antigen carrying DCs from site of antigen encounter to lymphoid organs, where these cells present the processed antigens to lymphocytes. During this activation associated migration process DC are known to down-regulate the expression of CCR5 and up-regulate the expression of CCR7. We also examined the effect of *in vitro* hyperthermia on the expression of these migratory markers by flow cytometry. Results are plotted in Figure 1. We observed a marked down-regulation in expression of CCR5 in immature BMDCs and up regulation of CCR7 in both maturing as well as immature cells, indicating that hyperthermia alone is capable of inducing DC migration in absence of any other maturation signal. These flow cytometry results indicate that *in vitro* hyperthermia promotes the phenotypic activation of DCs and also suggests its potential for enhancing activation associated migration of DCs *in vivo*.

### *In vitro* hyperthermia enhances T cell stimulatory capacity of dendritic cells in allo MLR

**Figure 2:**
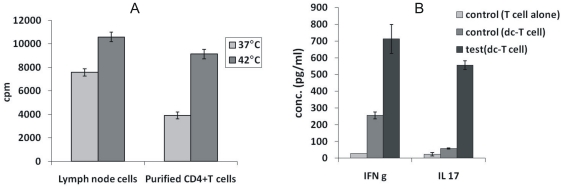
A. *In vitro* hyperthermia enhances the T cell proliferative capacity of BMDCs in allo MLR. BMDCs from Balb/c mice (stimulator; 0.1 M) were treated at 37°C or 42°C for 30 minutes and co-cultured with either lymph node cells or purified CD 4+ T cells from spleen. B. Cytokines secreted in supernatant of allo MLR as estimated by ELISA.

The ability to prime naïve T cells is a critical function of dendritc cells. Antigen pulsed DCs are known to elicit potent antigen specific T helper responses in mice [22]. To examine the functional maturation of BMDCs by hyperthermia, we compared the T cell stimulatory capacity of *in vitro* hyperthermia treated BMDCs (from Balb/c mice) to that of untreated BMDCs in an allo MLR. As shown in Figure 2A, we found a significant (*) increase (p = .0143) in the thymidine incorporation in cultures where treated BMDCs were compared with untreated cells as stimulator (0.1 M) cells to test their ability to stimulate responder (0.5 M) lymph node cells from C57BL/6. A significant (**) increase (p = 0.0086) in proliferative capacity was also observed when we used purified CD4+ T cells from spleen as responder cells (0.5 M). Culture supernatant was also collected after 48 h and checked for secretion of IFN-γ, IL-4 and IL-17. Figure 2B shows that while in vitro hyperthermia increased the secretion of both IFN-γ and IL-17, no detectable amount of IL-4 was secreted in the cultures. It is to be noted that the cultures comprising of untreated DCs also secretion IFN-γ and IL-17 albeit at lower levels than cultures comprising of treated samples. Also, no detectable IL-4 was observed even in control cultures. This result verifies that in vitro hyperthermia leads to maturation of BMDCs not only in terms of phenotypic expression of maturation markers, but also functionally in terms of their ability to stimulate T cells. These observations suggest that the use of hyperthermia *in vivo* may lead to better stimulation of T cells upon interacting with them in the lymphoid organs.

### *In vitro* hyperthermia enhances cytokine production by dendritic cells

**Figure 3:**
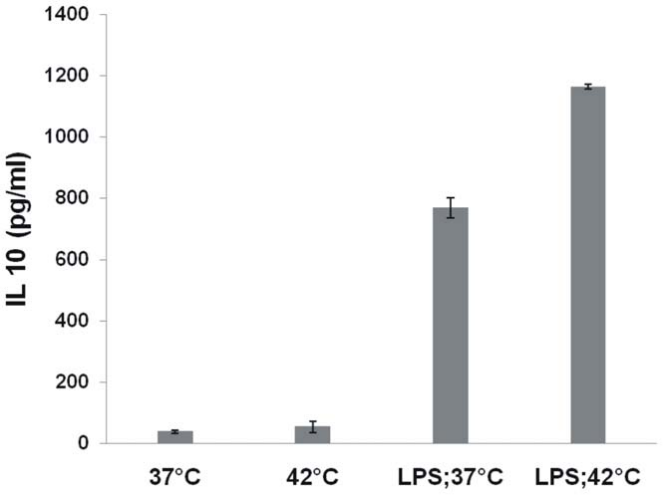
*In vitro* hyperthermia affects the secretion of IL-10 by BMDCs. Immature or LPS matured (1 µg/ml; 6 hour) BMDCs were cultured at either 37°C or 42°C for 30 minutes followed by recovery at 37°C. Supernatant were collected after 24 hours and cytokine levels were determined by sandwich ELISA. Results are plotted as Mean ±SD of a representative of three experiments.

**Figure 4:**
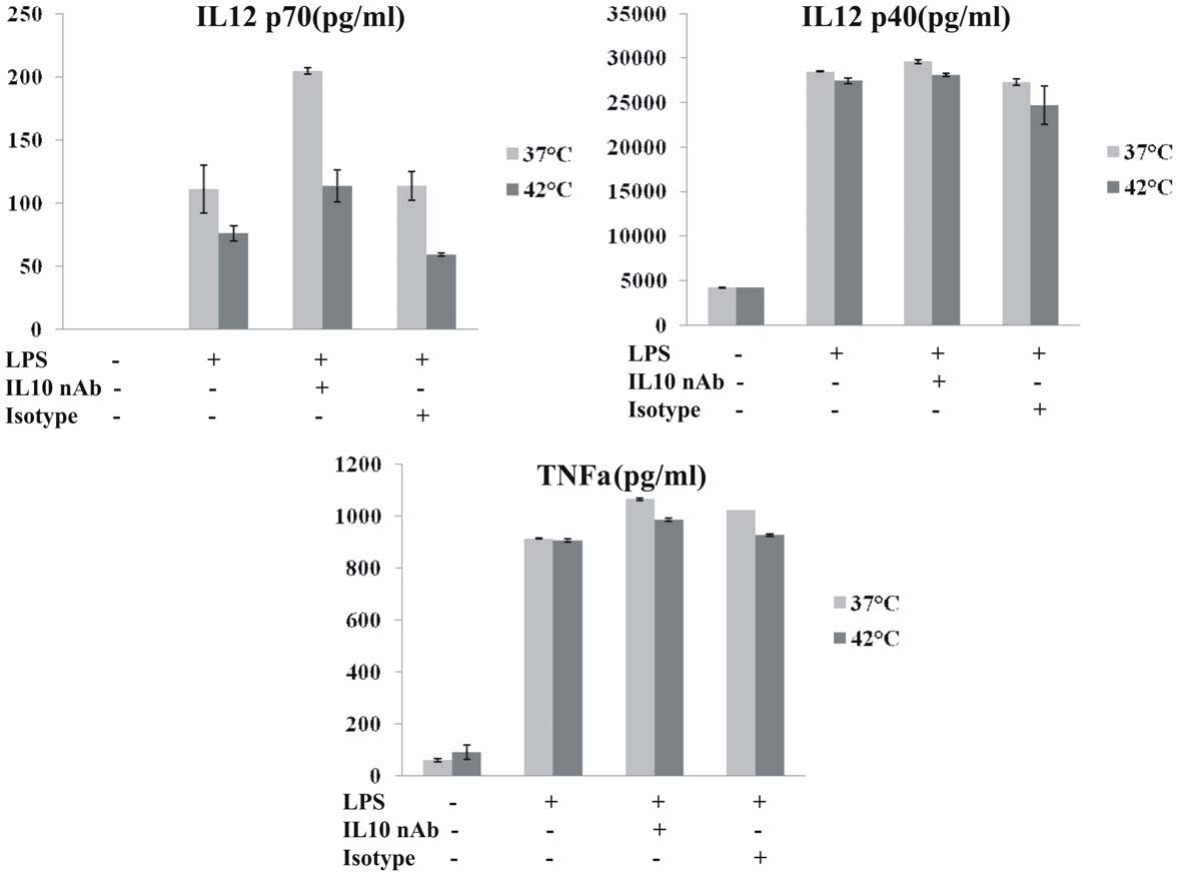
Effect of IL-10 neutralization on production of IL-12p70, IL-12p40 and TNF-α by DCs pretreated with in vitro hyperthermia. DCs were incubated at 37°C (light gray) or 42°C (dark gray) for 30 minutes, and allowed to recover at 37°C for 6 hours. Then 0.1 µg/ml anti-IL-10 mAb or isotype control was added at the time of stimulation with 1 µg/ml LPS at 37°C for additional 22 hours. The supernatants were collected and the concentrations of IL-12p70, IL-12p40 and TNF-α were measured by ELISA. Data are shown as Mean ±SD of one typical experiment of three independent experiments.

Upon antigen encounter and uptake, dendritic cells are known to undergo maturation during which they secrete various cytokines. The cytokine milieu in turn is an important factor governing the type of immune response that will be generated. So next, we studied the effect of *in vitro* hyperthermia on induction of cytokine secretion by BMDCs. The cytokines secreted by DCs contributes in determining the class of immune response by providing suitable microenvironment to T cells. While the production of IL-12 by DCs induces a type 1 pattern of cytokine secretion by T cells, higher IL 10 is known to promote a Th2 kind of Th cell response [23]. TNF-α is a plieotropic cytokine with diverse functions in inflammation. Immature DCs are not known to secrete these cytokines. As observed in Figure 3, while *in vitro* hyperthermia alone did not lead to induction of cytokine secretion by immature DCs, maturing DCs (matured by addition of LPS) were found to secrete higher levels of IL-10 but lesser amount of IL-12p70. The levels of TNF-α, however remained unchanged (Figure 4).

Next, we performed further experiments to investigate if the regulation of IL-12p70 and TNF-α secretion upon hyperthermia exposure, depends on IL-10 secretion. Autocrine secretion of IL-10 is known to affect the secretion of TNF-α as well as IL-12p70 due to its anti inflammatory property. To establish whether treatment by *in vitro* hyperthermia is incapable of stimulating the production of TNF-α and IL-12p70 and the observed decrease in case of IL-12p70 is not due to autocrine secretion of large amounts of IL-10, we performed neutralization experiment in which the IL-10 secretion in DC cultures was neutralized using IL-10 mAb. We found that the secretion of TNF-α and IL-12p40 remains unaffected after IL-10 neutralization.

IL-12p70 is a hetero-dimer of IL-12 p40 and IL-12p35 subunits. IL-10 secretion is known to down regulate with secretion of IL-12p35. Therefore upon IL-10 neutralization, more of IL-12p35 may be produced and higher level of IL-12p70 was detected. But this increase is independent of hyperthermia treatment as it was observed in both control (37°C) and hyperthermia group for TNF-α. Moreover the amount of IL-12p70 continues to be lesser in hyperthermia group even after neutralization of IL-10, thus establishing that *in vitro* hyperthermia indeed stimulates BMDCs towards IL-10 secretion. This may be helpful in skewing the immune response towards Th2 type.

### *In vivo* local hyperthermia enhances DC maturation and migration in draining lymph node

**Figure 5:**
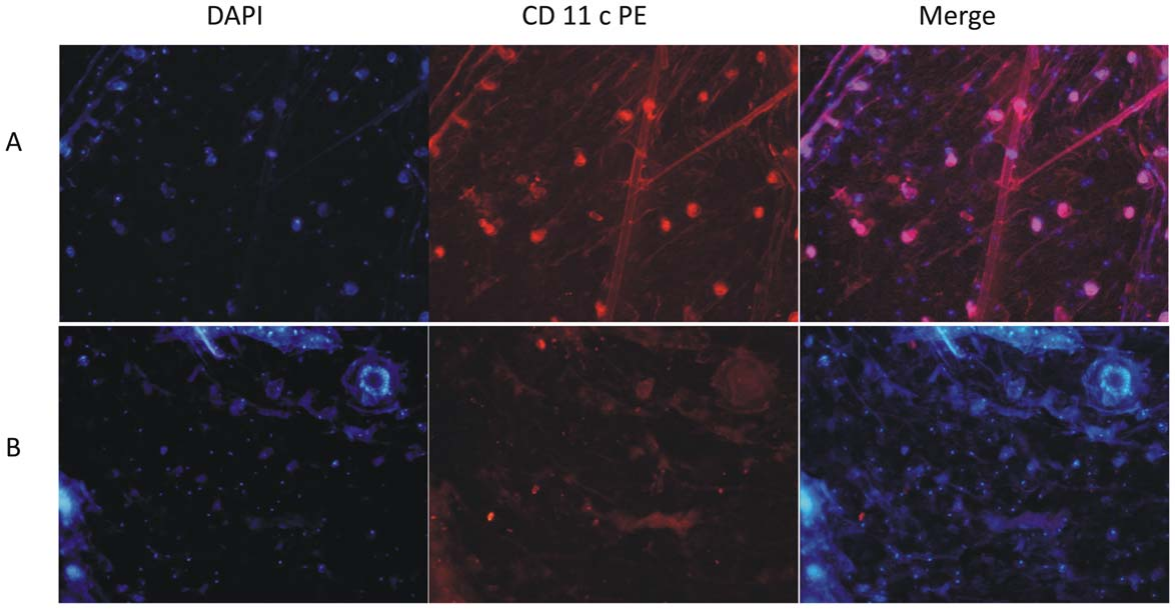
Migration of DCs from epidermis upon local Hyperthermia. Mice were given local hyperthermia by placing hyperthermia patched on shaved skin. Epidermal sheets were prepared from control (A) and treated (B) mice 24 hours after treatments and stained for CD11c (PE). Nuclear staining was done using DAPI. Images were taken at 10× and are representative of three independent experiments.

To determine the effect of local hyperthermia on dermal DC maturation and migration to regional lymph node, we subjected mice to *in vivo* local hyperthermia by placing the hyperthermia inducing patch, containing either PBS or antigen, on the right thigh of mice. After 24 hours, we prepared epidermal sheets from treated area as well as untreated area of the mice skin and stained them for CD11c+ cells. We observed a decrease in the number of CD11c+ (Figure 5) cells in epidermal sheets taken from area of skin where hyperthermia patch was placed, suggesting that short duration hyperthermia indeed causes migration of DCs from skin to lymph node.

**Figure 6:**
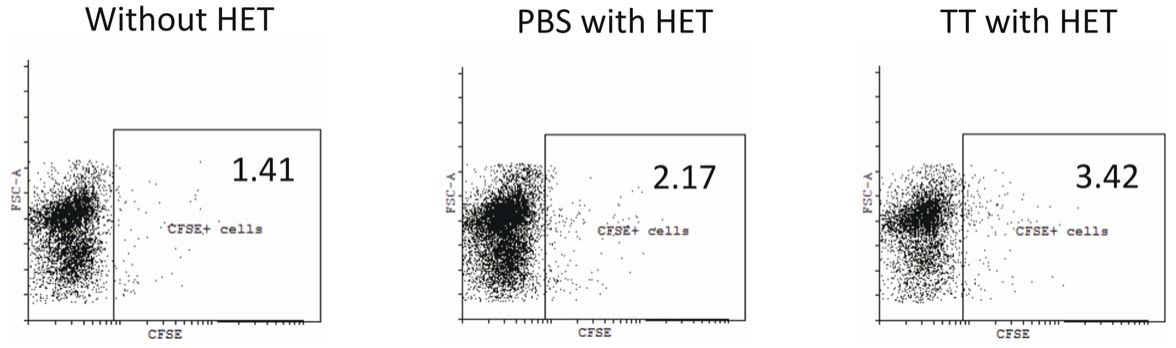
Migration of carboxyfluorescein diacetate succinimidyl ester (CFSE) stained skin DCs to the lymph node. DCs were fluorescently labelled *in vivo* by the cutaneous injection of CFSE (without HET) and in another experiments HET patch containing PBS or TT was placed at the site of CFSE injection. After 24 h, cells from draining lymph nodes were stained with CD11c-PE.Cy5 antibody. Typical dot plots are shown in which CD11c+ cells were gated and the CFSE+ cells were analyzed. Number on the selected area indicates percent of CFSE+ cells.

To further verify this, skin DCs were fluorescently labelled *in vivo* by the cutaneous injection of carboxyfluorescein diacetate succinimidyl ester (CFSE) [24] and HET patch was placed at the site of CFSE injection. We prepared the lymph node suspensions after 24 hours of treatment, stained them with CD11c-PE.Cy5 antibody. The population was gated for CD11c+ cells on the basis of CD11c-PE.Cy5 staining and checked for the presence of CFSE+ cells, indicating the cells that have migrated from skin. Figure 6 shows that HET treatment leads to the enhanced migration of CD11c+ DCs from skin and the migration is further enhanced when an antigen (TT in our case) is incorporated in the HET patch.

**Figure 7:**
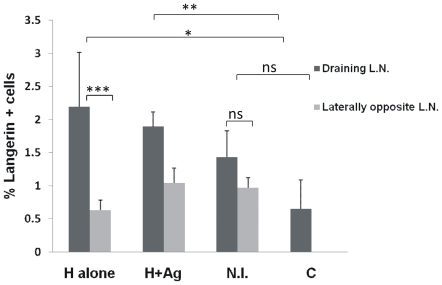
Migration of LCs from epidermis upon local Hyperthermia vs. needle immunization. Mice (n = 3) were immunized with TT by either conventional i/m (NI) or HET with (H+TT) or without (H) antigen. Unimmunized animals served as control. Cell suspension from draining lymph nodes were prepared after 24 h and analyzed by flow cytometry after staining with Langerin (CD207)–PE antibody. Results are expressed as % of positive cells in total draining lymph node population.

We also performed similar experiment with animals immunized by HET (along with TT antigen) and conventional needle immunization. We found (Figure 7) that compared to control, both HET groups (with or without antigen) showed a significant increase in expression of Langerin marker, thus indicating that mild hyperthermia of the HET patch is sufficient to cause an enhanced migration of DCs from skin to draining lymph node.

**Figure 8:**
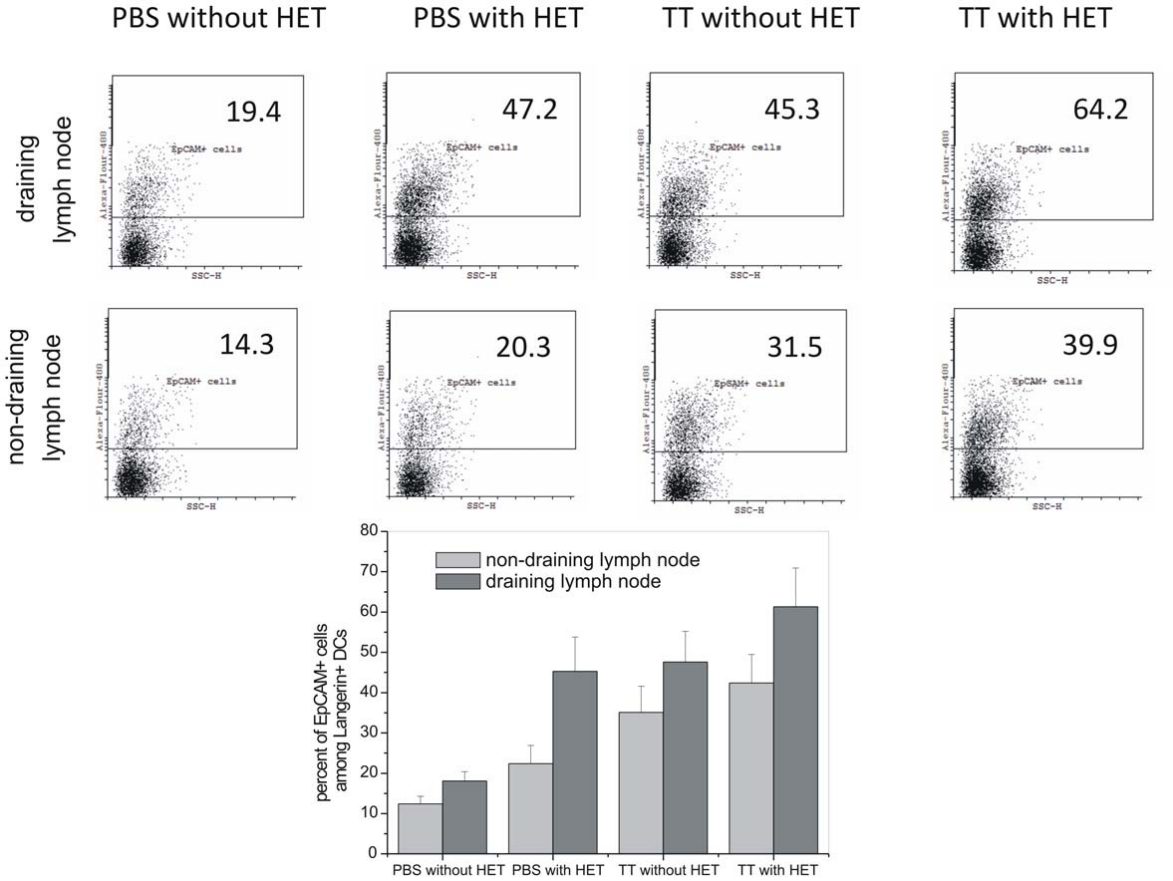
Migration of EpCAM+ epidermal DCs to the lymph node. Mice were immunized by TT, with and without HET. Unimmunized (PBS without HET) animals served as control. Cell suspension from draining and non-draining lymph nodes were prepared after 24 h and stained with Langerin (CD207)-PE and EpCAM-Alexa Flour 488 antibodies. Stained cells were analyzed by flow cytometry, Langerin+ cells were gated and the expression of EpCAM was analyzed. Number on the selected area indicates percent of EpCAM+ cells among Langerin+ cells in total lymph node cells. The bar graph shows average percentages of three experiments.

To distinguish epidermal DCs (LCs) among the Langerin+ dermal DCs present in the lymph node, we utilized the selective expression of cell adhesion molecule EpCAM on epidermal DCs [25]–[28]. Figure 8 shows the dot plots of Langerin+ gated cells. These data reinforce that heat alone leads to enhanced migration of epidermal, EpCAM+ DCs to the draining lymph node. The presence of an antigen (TT in our case) in the HET patch further added to this enhancement. Further, the enhanced migration remain localized to the nearest draining lymph node.

**Figure 9:**
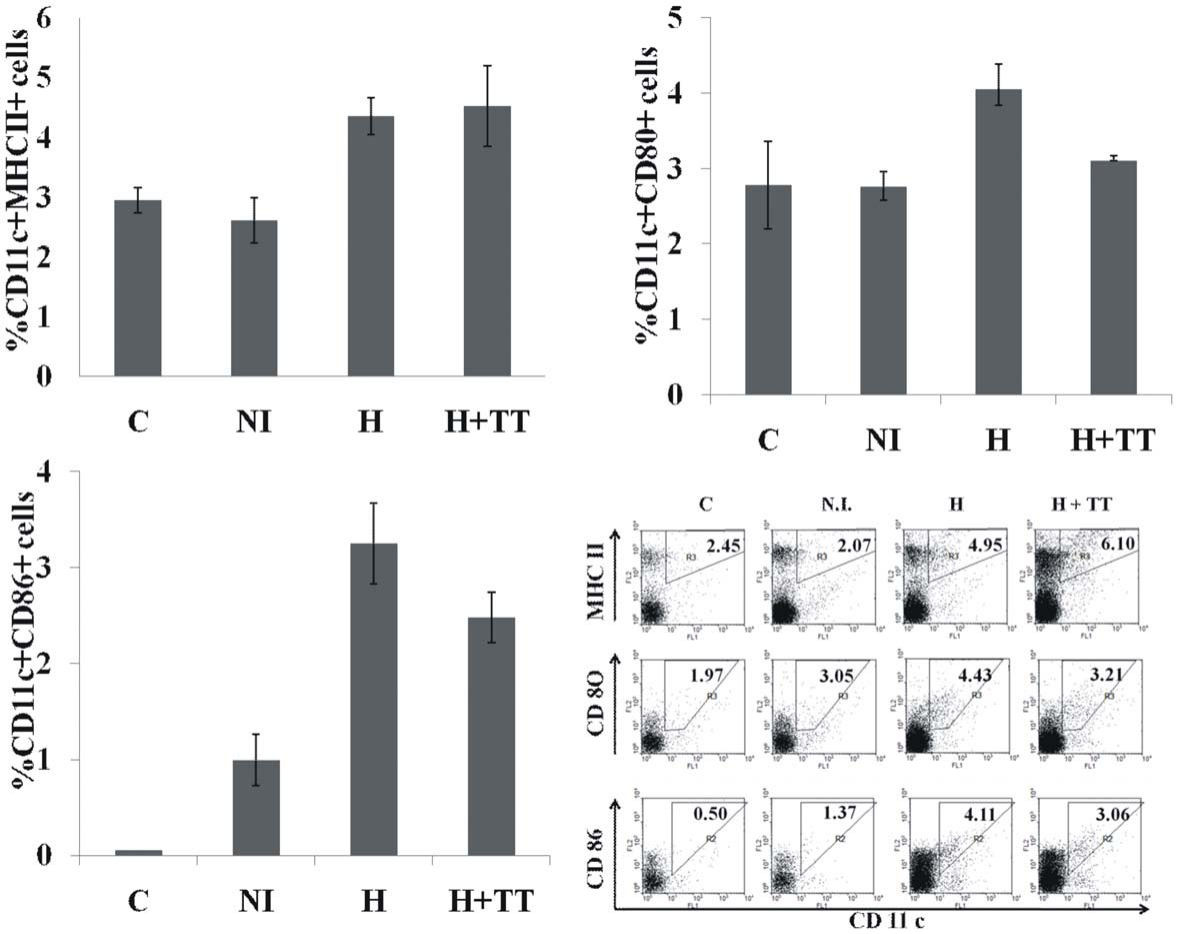
Immunization via intramuscular route and HET differentially affects the expression of MHC-II, CD 80 and CD 86 by DCs from draining lymph nodes. Mice (n = 3) were immunized with TT by either conventional i/m (NI) or HET with (H+TT) or without (H) antigen. Unimmunized animals served as control. Cell suspension from draining lymph nodes were prepared on day 2 and analyzed by flow cytometry after double staining with anti-CD 11c and anti-MHCII or anti-CD80 or anti-CD86. Results are expressed as % of double positive cells in total draining lymph node population. These results are a representative of three independent experiments.

We further compared the HET immunization technique to conventional needle immunization in terms of expression of maturation markers on DCs in draining lymph node upon immunization. Mice (n = 3) were immunized by either conventional intramuscular route or HET (with or without antigen). Unimmunized animals served as control. As seen in Figure 9, the expression of maturation marker on DCs of pooled draining lymph node was higher in both groups of hyperthermia as compared to needle immunization. Thus HET is not only superior to needle immunization in this respect, but the effect also seems to be independent of the presence of antigen, thus suggesting that thermal component of HET immunization may be responsible for this effect. This can be explained by our previous observation that *in-vitro* hyperthermia is able to enhance the expression of maturation and migration markers in BMDCs. Thus, the thermal component of HET serves as an adjuvant in promoting the maturation and migration of DCs to lymph node.

### Priming by HET generates memory response

**Figure 10:**
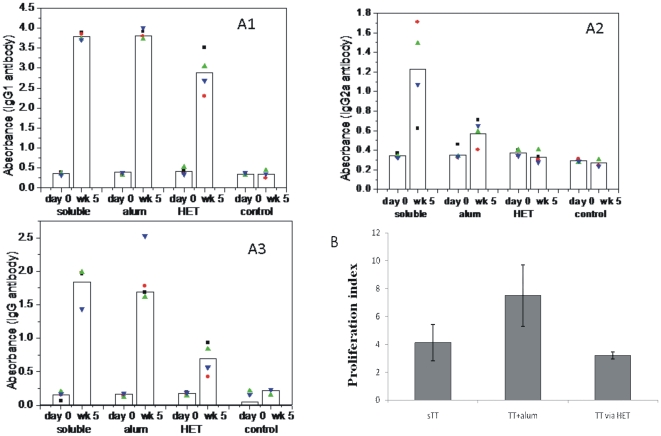
Serum anti-TT Abs in mice primed by HET. Mice were immunized by soluble TT alone (i/m), TT with alum (i/m) or TT via HET. Unimmunized animal served as control. Booster of soluble TT (i/m) was given on Day 28 and mice were bled on week 5. Serum anti TT IgG1 (A1), IgG2a (A2) and IgG (A3) were estimated by ELISA. Recall response was evaluated by estimating the proliferative index by *in vitro* restimulation of splenocytes from immunized animals with TT antigen (B). Each dot represents individual animal and bars represent the geometric mean of each group.

To evaluate the adjuvant effect of thermal component of HET on final immune outcome in terms of antibody production and recall response, we compared it with conventional alum adjuvant during immunization. Mice (n = 4) were immunized using soluble TT (sTT), TT with alum or TT via HET. A group of unimmunized mice (n = 3) served as control where no primary immunization was done. On day 28 after first immunization, booster was given to all the groups via i/m injection of TT without alum. One week later, the total serum IgG levels were estimated by ELISA. As seen in the Figure 8 panel A3, an increase is seen in total serum IgG levels in all immunization groups when compared with control. Though the levels are lower in HET group compared to sTT and TT with alum group, it is significantly higher than the control group, thus establishing the ability of HET immunization to generate a recall response. This is further established by the proliferation assay. One week after the booster dose, the proliferation response of cells from spleen were examined in which re-stimulation was done with TT. The results are depicted in Figure 10, panel B. The HET immunization led to the generation of proliferative response which is not significantly different (p = 0.1480) from TT with alum group. Thus HET immunization by TT is comparable to needle immunization for primary antigen administration in terms of generation of recall response. Further, to evaluate the immune modulatory potential of thermal component of hyperthermia, we performed the subclass analysis for serum IgG abs to TT, as the secretion of IgG1and IgG2a isotypes of immunoglobulins is hallmark of Th2 and Th1 phenotypes of immune responses, respectively [29]. As shown in the Figure 10, panel A1 and A2, while immunization with TT without alum leads to generation of both IgG1 and IgG2a abs suggesting a mixed Th response, the response generated in presence of alum (TT with alum) or by HET was clearly skewed to Th2 type with higher serum IgG1 compared to IgG2a.This observation highlights that similar to the use of alum as adjuvant [30], HET is also able to selectively potentiate a Th2 kind of response. We have previously shown (Figure 3) that the thermal component of HET stimulate the DCs to secrete IL-10 *in vitro*. It is known that IL-10 play an important role in the Th2 response to antigen [31], it thus provides a possible explanation for Th2 skew during immunization by HET.

### HET immunization protects mice against challenge

**Figure 11:**
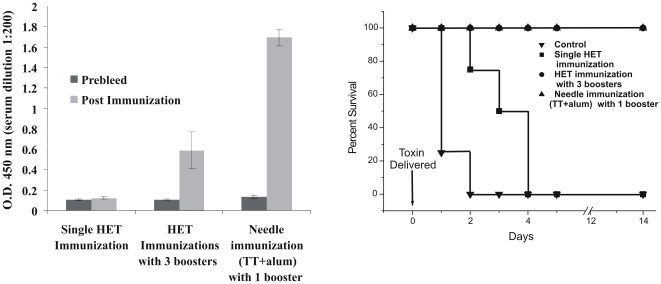
Boosting upon HET immunization and toxin challenge. Mice were immunized via a single dose of HET patch or multiple doses of HET patch on day 0, 14, 28 and 42. The positive control was needle immunized with TT+alum on day 0 and 28. Toxin challenge was given one week after the completion of immunization schedule.

The success of any vaccine therapy depends on its ability to confer protection against actual biological challenge. Thus to determine the value of HET immunization as a novel transdermal vaccine delivery technique, we wanted to evaluate its protective efficacy. Since in our immunization experiments we found that HET elicits lower IgG levels compared to convention i.m. immunization, we followed multiple immunization regimen using higher concentration of antigen (200 µg TT in the HET patch). Mice (n = 4) were immunized with 4 doses of TT via HET (Day 0, Day 14, Day 28 and Day 42). Serum IgG levels were determined one week after the last antigen dose and compared with single dose HET immunization as well as i.m. needle immunization with TT along with alum (single booster), as seen in Figure 11. One week after the completion of immunization schedule, mice were challenged with LD_50_ of biologically active toxin and observed for a period of 5 days for mortality or paralysis. All animals in the control group (unimmunized) were dead by the end of day 2. The single dose of HET immunization was unable to provide sufficient protection and all the animals were dead by the 4th day. In the multiple HET immunization test group and conventional needle immunization with one booster group, no death or paralysis was observed and all the animals survived. The mice in the test group did not show any symptoms of disease even after 2 weeks of challenge, thus establishing the protective efficacy of vaccine delivered through HET.

## Discussion

The present study demonstrates that HET immunization is an efficient way of needle free immunization generating a noticeable serum antibody as well as recall response against the administered antigen. Besides facilitating the passage of antigen across the skin, the thermal component also induces the maturation and migration of dendritic cells from the site of antigen application to the draining lymph node. Moreover, similar to conventional adjuvants like alum, HET also favours the development of IgG1 antibody isotype upon recall, thus favouring a Th2 kind of immune response.

The finding of this study that mild heat shock causes maturation of DCs is in agreement with previously published reports [32]–[34]. Basu et al. [35] have investigated the effect of mild thermal stress (up to 40°C) on the maturation of DCs and reported that the duration of hyperthermia as well as the recovery at 37°C is critical for the up regulation of maturation markers. On the contrary there is report by Tournier et al. [36] which shows that thermal stimuli for long durations of 3 hours with recovery period of 15 hours at 37°C had no effect on DC maturation. In the present study, we have observed that hyperthermia at 42°C for a short duration of 30 minutes is also able to cause up regulation of DC maturation markers. Moreover, the levels of MHC-II were found to be elevated within 2 hours after treatment. While the co-stimulatory molecules peaked at 24 hours of recovery, the migratory markers showed maximum differential expression in 36 hours. This study thus highlight that the optimum recovery period depends on the marker maturation being investigated. This knowledge is critical to observe the desirable effect of hyperthermia protocol being investigated and should comprise an essential parameter of all investigations in this field.

Mature DCs are the most potent APCs for stimulating naïve T cells. Thus examining the T cell stimulatory capacity is physiologically most significant feature for DC maturation. Our observation that hyperthermia is able to enhance allo-stimulatory T cell capacity is in agreement with other reports where the enhancement in allo-stimulation at different combinations of temperature and time for stimulation was investigated [33]–[36].

Besides up regulation of maturation markers, DC maturation is accompanied by synthesis of inflammatory cytokines. Our observation that hyperthermia alone does not induce cytokine secretion by immature DCs is in accordance with previously published work [36], but after the hyperthermia exposure at 42°C, the DCs matured by LPS treatment show a cytokine profile which is distinct from previous reports. While hyperthermia at temperatures up to 40°C is known to enhance secretion of pro-inflammatory cytokine IL-12p70 and reduce the secretion of IL-10 and TNF-α [36], upon treatment at 42°C in our study, we found the down regulation in IL-12p70 and the level of IL-10 was slightly elevated. The level of TNF-α remains unchanged. This observation suggests that the mechanism underlying DC maturation upon thermal stimulation may vary across the different temperature ranges. Whereas the BMDCs maturation induced by mild thermal stress have been reported to be independent of heat shock associated transcription factor Hsf-1 [34], [37], at temperatures over 40°C heat shock proteins are usually induced which can regulate cytokine expression by terminating general protein synthesis or by inhibiting the transcription of cytokine genes. Similar observations have also been reported on macrophages [38]. Thus to accurately predict the outcome of thermal stimuli on immune outcome, it is necessary to establish the mechanism by which hyperthermia regulates DCs maturation across various temperature ranges. This would comprise the most important area of future research in hyperthermia mediated DC maturation.

Transcutaneous immunization is known to cause an enhanced migration of skin dendritic cells from the topical site to the draining lymph nodes [39], [40]. Our results are also concurrent with these observations, but unlike other published studies, we have shown that the enhanced migration can be induced by hyperthermia alone even in absence of antigen. We have demonstrated *in vivo* that the hyperthermia causes activation associated migration in skin dendritic cells. Furthermore, hyperthermia not only contributes to the enhanced migration of epidermal DCs, it also increases the kinetics with the migration peaking in 24 hours as compared to 2–4 days required by conventional needle immunization (unpublished data). Thus the thermal component of HET indeed plays an important role in modulating the maturation and migration of skin DCs.

Secretion of IgG1 and IgG2a isotypes of immunoglobulins is hallmark of Th2 and Th1 phenotypes of immune responses, respectively [29]. Conventional adjuvants like alum show immune modulatory function by promoting the production of IgG1 isotype, thus promoting a Th2 kind of immune response [41]. We also observed that upon giving a booster, the animals those received TT by HET or conventional immunization along with alum showed more of IgG1 kind of isotype whereas animals immunized by soluble TT alone produced a mix response showing both IgG1 as well as IgG2a kind of serum isotypes. Thus the thermal component of hyperthermia acts similar to the alum adjuvant by promoting the production of Th2 isotype ultimately to produce the Th2 skew.

The merits of using HET immunization are quite apparent in being a painless, needle free delivery system. Moreover, since it has significant immune modulatory potential, it will be interesting to study this mode of immunization in context of poor immunogens such as polysaccharides and haptens. But, the feasibility for its clinical application will depend on the availability of a method for accurate estimation of dose delivered, and currently we are focusing on various strategies towards achieving this goal.

## Materials and Methods

### Ethics statement

All the procedures on animals were approved by the Institutional Animal Ethics Committee of the National Institute of Immunology, project serial number IAEC#/204/08.

### Mice and immunization

BALB/c and C57BL/6 mice (The Jackson Laboratory, Bar Harbor, ME), bred in the small animal facility of the National Institute of Immunology, New Delhi, India were used in all experiments. Mice were immunized intramuscularly with 2 µg/animal of TT Ag (C.R.I., Kasauli) with or without alum 2 µg/animal (Alhydrogel, Vedback, Denmark) in the needle immunization group. In the HET immunization group, 20 µg of soluble antigen (in PBS)/animal was placed in the immunization patch at 42°C for 30 minutes as previously mentioned [11]. Briefly, for HET immunization, mice were shaved on day -1 using cosmetic grade hair removal cream and immunized on day 0 by placing hyperthermia patch filled with 200 µl of antigen solution on the shaved area for 30 minutes. Additional details on the HET immunization procedure are given in Figure S1. Mice were bled from the retro-orbital venous plexus under ketamine/xylazine anaesthesia and sera separated for Ab assays. For lymph node cells activation markers expression, DCs migration, and measurement of T cell responses, mice were euthanized by cervical dislocation and spleens or lymph nodes were isolated and single cell suspensions were prepared for use. Group sizes of 3–5 mice each were used for the serologic experiments, while 3 mice each were used for the T cell proliferation and activation and migration analysis experiments.

### Generation of bone marrow dendritic cells (BMDCs)

BMDCs were generated *in vitro* from bone marrow. Briefly, femur and tibiae were removed from 8–10 weeks old Balb/c mice. Intact bones were left in 70% ethanol for 1 minute for disinfection and washed with PBS. Both the ends of bone were cut open under sterile conditions, bone marrow cells were flushed out and washed with RPMI1640 medium. Bone marrow cells (4×10^6^) were cultured in 6 well plates at concentration of 10^6^ cells/ml in RPMI 1640 (Biological Industries) medium supplemented with 10% heat inactivated FBS (Biological Industries), 100 Units/ml penicillin, 100 µg/ml streptomycin, 250 µg/ml amphotericin B (Sigma) and 10 ng/ml murine rGM-CSF (Peprotech) at 37°C and 5%CO_2_. On day 3 and 5, 75% of media was removed from each well and 3 ml of fresh media supplemented with 10 ng/ml GM-CSF was added. On day 7, non adherent cells were harvested and used as immature DCs. The immature DCs were given a hyperthermia treatment at 42°C for 30 minutes followed by recovery at 37°C for indicated time period. The control cells were maintained at 37°C during this treatment period.

### *In vivo* Local Hyperthermia and kinetics of DC migration, antigen presenting and co-stimulatory activity in lymph node

To determine the effect of local hyperthermia on the kinetics of DC migration and MHCII and co-stimulatory molecule expression in lymph node, animals were subjected to 30 minutes of local hyperthermia by placing the patch (PBS alone or PBS containing antigen) on the right thigh of the animal. Inguinal lymph nodes from the same side as well as laterally opposite side were isolated after indicated time periods (24, 48 and 72 hours). Cells harvested from the lymph node were examined for expression of Langerin, EpCAM, MHCII, CD80, CD86, and CD11c markers by flow cytometry.

For the *in vivo* staining of endogenous skin DCs, mice were given cutaneous injection of CFSE (Molecular Probes), 80 µg/animal in PBS at the shaved area of flank under anesthetic conditions. After the 15 minutes of CFSE injection mice were subjected to HET treatment.

### Flow cytometry

For flow cytometry, cells (5×10^5^/well) were incubated with 1 µg/ml anti-mouse CD16/32 antibodies (BD biosciences) in the blocking buffer (0.1% sodium azide +1%BSA in PBS) for 30 minutes at 4°C to block non specific binding to FcγIII/II receptor. After washing with 0.1% sodium azide +1% BSA in PBS, cells were incubated with primary antibodies (in PBS with 1% BSA) on ice for 1 hour. After washing three times, cells were incubated with secondary antibodies on ice for 1 hour. Stained cells were washed thrice before fixing with 4% paraformaldehyde and stored at 4°C until analyzed. Samples were run on a BD LSR flow cytometer and data were analyzed using WinMDI 2.8 or Cyflogic 1.2.1 shareware. Anti-CD11C-FITC (eBiosciences), was used as the surface marker of DCs and Biotinylated-MHC-II, Biotinylated-CD80, and Biotinylated-CD86 (all from BD Biosciences) were used as primary antibodies, whereas S-PE (BD-Biosciences) was used as secondary antibody.

For the analysis of *in vivo* CFSE stained skin DCs in the lymph node, cells obtained from the lymph node were stained with CD11c-Biotin (BD-Biosciences) (S-PE.Cy5 (eBiosceince) as secondary Ab). Because the number of CFSE+ cells was very small, we ‘gated in’ only the CD11c+ cells and examined the CFSE+ cells.

Biotin-EpCAM (eBiosceince) mAb - Streptavdin-Alexa Flour 488 (Invtrogen) and PE conjugated Langerin (CD207) (eBiosceince) mAb were used to distinguish epidermal DCs among Langerin+ cells in the lymph node. Since the number of Langerin+ cells is very small (typically 0.5%) in the lymph node, it was difficult to visualize minor variations in the number of double positive (Langerin+, EpCAM+) cells among different groups in a conventional dot plot. We thus ‘gated in’ only the Langerin+ cells and selected the EpCAM+ cells.

### Allogenic Mixed Lymphocyte Reaction (MLR)

To estimate the proliferative capacity of BMDCs, DCs were cultured from Balb/c mice and exposed to *in vitro* hyperthermia. DCs from the hyperthermia group and the control were then co-cultured with total lymph node cells or magnetically purified CD4+ cells from spleen of C57BL/6 mouse in the allo MLR to determine their proliferative capacity by estimating the incorporation of tritiated thymidine in the proliferative cells. Briefly, the DCs (stimulator) and lymph node cells or purified CD4 T cells (responder) cells were co-cultured at stimulator: responder concentrations of 1∶5 in 96-well U bottom microtiter plates (Costar-Corning). Tritiated thymidine (0.5 µCi/well) was added at 72 hours and plates were frozen after 18 hours of thymidine addition. For measuring the incorporated radioactivity, plates were harvested on printed filtermats (Wallac) by micro 96 harvester (Molecular Devices) and counts measured using β-scintillation counter (Perkin Elmer).

### IL10 neutralization

BMDCs harvested at Day 7 of the culture were plated at concentration of 1 M cells/ml in a 24 well plate. For performing IL 10 neutralization, DC were either kept at 37°C (control) or exposed to 42°C (hyperthermia) for 30 minutes and allowed to recover at 37°C for 6 hours. After the recovery, 0.2 µg/ml of neutralizing antibody (Anti mouse IL 10, clone JES5-245, eBiosciences) or isotype control were added. After 1 hour of incubation with neutralizing antibodies, LPS was added at 1 µg/ml concentration. Supernatant was collected after 22 hours of culture and stored at −70°C till assay was performed.

### Cytokine measurement in supernatants

Cytokine levels in supernatants were determined by using commercial sandwich ELISA kits. IL-12p70 ELISA was performed using eBiosciences kit whereas IL-10, IL-12 p40, and TNF-α ELISA were performed using kit from BD Biosciences. The ELISA reactions were developed using TMB substrate (BD biosciences) and read at 450 nm.

### Immunohistochemistry of skin

Balb/c mice were shaved using cosmetic grade hair removing cream. Next day mice were given local hyperthermia. After defined period of time, mice were euthanized and skin from the shaved area was cut using scissors. The skin sample was then placed in ice cold 0.5% dispase solution, dermal side down taking precaution to avoid any curling. The skin sample was then incubated at 4°C for 24 hours. Skin was then placed epidermal side down on a slide and spread evenly to avoid any curling. It was incubated at 37°C for 1 minute to allow the epidermis to adhere to culture dish. Using fine forceps, the dermis was gently lifted to allow only epidermis to stick. The epidermis was fixed using 4% paraformaldehyde solution at room temperature (25°C) for 30 minutes. It was then washed with PBS. Blocking was done by 1% BSA in PBS for 30 minutes. Primary antibody CD11c-biotin was added and incubated at room temperature for 1 hour. The sample was washed three times with PBS, followed by addition of streptavidin-PE. The sample was further incubated at room temperature for 1 hour followed by three washes with PBS. Sample was then incubated with DAPI for 5 minutes followed by three PBS washes. Finally the sample was mounted using glycerol, covered with a cover slip and observed under florescent microscope (Olympus IX 51) and images were captured using DP controller software.

### Ag-specific Ab assays

Antigen specific antibodies in immunized animal were detected using indirect ELISA. Briefly, Maxisorp microtiter plates (Nunc) were coated with the TT (1 µg/ml). Abs in immune sera were detected by enzyme immunoassays using goat anti-mouse total IgG (Sigma), IgG1 and IgG2a peroxidase (Serotec) followed by TMB substrate solution (BD biosciences) and reaction was stopped using 2N sulphuric acid. Optical density at 450 nm were observed and used for comparing serum antibody levels.

### Proliferation Assays

The memory response in the immunized animals was determined using T cell proliferation assay. Briefly, one week after the booster dose splenocytes were derived from animals in all the groups. The splenocytes were then plated at concentration of 0.5 M cells/well in a 96 well cell culture plate, followed by addition of 0.1 µg/well of TT. Tritiated thymidine was added after 72 hours. After another 18 hours, plates were frozen at −70°C until the assay was performed.

### Toxin challenge

Mice groups (n = 4) injected s.c. 0.1 ml of different dilutions of tetanus toxin (Indian Immunologicals, Hyderabad). Mice were observed twice daily for 5 days for death/paralysis. LD_50_ was defined as the dose at which at least 50% animals die/develop paralysis by the end of 5 days and was determined to be 4.6 µg/animal. Test and control mice (n = 4) were challenged s.c. with LD_50_. Mice were observed daily following challenge, and both morbidity and mortality were recorded.

### Statistical analysis

Statistical analysis was done using the two sided Student's *t*-test. Data are presented as mean ± SD. The statistical analysis was performed using Graph Pad Instat software (GraphPad Software Inc. version 3.05).

## Supporting Information

Figure S1 The confirmation of protocol for local hyperthermia. Panel A shows the surgical implantation of the thermistor probe in the mice and panel B depicts how the heating patch to generate hyperthermia was placed over the skin. Panel C shows the temperature profile generated upon treating the mice with local hyperthermia at 42°C which confirms that the temperature beneath skin immediately attains the desired temperature.
(TIF)

Figure S2 Kinetics of expression of CD40 on BMDCs. Hyperthermia enhances the expression of CD 40 maturation marker on DCs. BMDCs were given *in-vitro* hyperthermia (42°C, 30 minutes) with or without LPS maturation. In absences of hyperthermia the expression of marker peaks after 48 hours whereas upon hyperthermia stimulation, the peak appears at 36 hours of LPS stimulation.
(TIF)

## References

[1] Mitragotri S (2005). Immunization without needles.. Nat Rev Immunol.

[2] Vandermeulen G, Staes E, Vanderhaeghen ML, Bureau MF, Scherman D (2007). Optimisation of intradermal DNA electrotransfer for immunisation.. J Control Release.

[3] Mitragotri S, Blankschtein D, Langer R (1995). Ultrasound-mediated transdermal protein delivery.. Science.

[4] Ogura M, Paliwal S, Mitragotri S (2008). Low-frequency sonophoresis: current status and future prospects.. Adv Drug Deliv Rev.

[5] Ding Z, Verbaan FJ, Bivas-Benita M, Bungener L, Huckriede A (2009). Microneedle arrays for the transcutaneous immunization of diphtheria and influenza in BALB/c mice.. J Control Release.

[6] Zhu Q, Zarnitsyn VG, Ye L, Wen Z, Gao Y (2009). Immunization by vaccine-coated microneedle arrays protects against lethal influenza virus challenge.. Proc Natl Acad Sci U S A.

[7] Karande P, Jain A, Ergun K, Kispersky V, Mitragotri S (2005). Design principles of chemical penetration enhancers for transdermal drug delivery.. Proc Natl Acad Sci U S A.

[8] Karande P, Arora A, Pham TK, Stevens D, Wojicki A (2009). Transcutaneous immunization using common chemicals.. J Control Release.

[9] Baxter J, Mitragotri S (2006). Needle-free liquid jet injections: mechanisms and applications.. Expert Rev Med Devices.

[10] Schramm-Baxter J, Mitragotri S (2004). Needle-free jet injections: dependence of jet penetration and dispersion in the skin on jet power.. J Control Release.

[11] Upadhyay P (2006). Enhanced transdermal-immunization with diptheria-toxoid using local hyperthermia.. Vaccine.

[12] Evans SS, Wang WC, Bain MD, Burd R, Ostberg JR (2001). Fever-range hyperthermia dynamically regulates lymphocyte delivery to high endothelial venules.. Blood.

[13] Ostberg JR, Patel R, Repasky EA (2000). Regulation of immune activity by mild (fever-range) whole body hyperthermia: effects on epidermal Langerhans cells.. Cell Stress Chaperones.

[14] Ostberg JR, Taylor SL, Baumann H, Repasky EA (2000). Regulatory effects of fever-range whole-body hyperthermia on the LPS-induced acute inflammatory response.. J Leukoc Biol.

[15] Mukhopadhaya A, Mendecki J, Dong X, Liu L, Kalnicki S (2007). Localized hyperthermia combined with intratumoral dendritic cells induces systemic antitumor immunity.. Cancer Res.

[16] Schueller G, Stift A, Friedl J, Dubsky P, Bachleitner-Hofmann T (2003). Hyperthermia improves cellular immune response to human hepatocellular carcinoma subsequent to co-culture with tumor lysate pulsed dendritic cells.. Int J Oncol.

[17] Kissenpfennig A, Henri S, Dubois B, Laplace-Builhe C, Perrin P (2005). Dynamics and function of Langerhans cells in vivo: dermal dendritic cells colonize lymph node areas distinct from slower migrating Langerhans cells.. Immunity.

[18] Sallusto F (2001). Origin and migratory properties of dendritic cells in the skin.. Curr Opin Allergy Clin Immunol.

[19] Cella M, Sallusto F, Lanzavecchia A (1997). Origin, maturation and antigen presenting function of dendritic cells.. Curr Opin Immunol.

[20] Jiang HR, Muckersie E, Robertson M, Xu H, Liversidge J (2002). Secretion of interleukin-10 or interleukin-12 by LPS-activated dendritic cells is critically dependent on time of stimulus relative to initiation of purified DC culture.. J Leukoc Biol.

[21] Pulendran B (2004). Modulating TH1/TH2 responses with microbes, dendritic cells, and pathogen recognition receptors.. Immunol Res.

[22] Banchereau J, Briere F, Caux C, Davoust J, Lebecque S (2000). Immunobiology of dendritic cells.. Annu Rev Immunol.

[23] Moser M, Murphy KM (2000). Dendritic cell regulation of TH1-TH2 development.. Nat Immunol.

[24] Miller MJ, Hejazi AS, Wei SH, Cahalan MD, Parker I (2004). T cell repertoire scanning is promoted by dynamic dendritic cell behavior and random T cell motility in the lymph node.. Proc Natl Acad Sci U S A.

[25] Nagao K, Ginhoux F, Leitner WW, Motegi S, Bennett CL (2009). Murine epidermal Langerhans cells and langerin-expressing dermal dendritic cells are unrelated and exhibit distinct functions.. Proc Natl Acad Sci U S A.

[26] Borkowski TA, Nelson AJ, Farr AG, Udey MC (1996). Expression of gp40, the murine homologue of human epithelial cell adhesion molecule (Ep-CAM), by murine dendritic cells.. Eur J Immunol.

[27] Bursch LS, Wang L, Igyarto B, Kissenpfennig A, Malissen B (2007). Identification of a novel population of Langerin+ dendritic cells.. J Exp Med.

[28] Ginhoux F, Liu K, Helft J, Bogunovic M, Greter M (2009). The origin and development of nonlymphoid tissue CD103+ DCs.. J Exp Med.

[29] Stevens TL, Bossie A, Sanders VM, Fernandez-Botran R, Coffman RL (1988). Regulation of antibody isotype secretion by subsets of antigen-specific helper T cells.. Nature.

[30] Li H, Willingham SB, Ting JP, Re F (2008). Cutting edge: inflammasome activation by alum and alum's adjuvant effect are mediated by NLRP3.. J Immunol.

[31] Laouini D, Alenius H, Bryce P, Oettgen H, Tsitsikov E (2003). IL-10 is critical for Th2 responses in a murine model of allergic dermatitis.. J Clin Invest.

[32] Ostberg JR, Gellin C, Patel R, Repasky EA (2001). Regulatory potential of fever-range whole body hyperthermia on Langerhans cells and lymphocytes in an antigen-dependent cellular immune response.. J Immunol.

[33] Ostberg JR, Kabingu E, Repasky EA (2003). Thermal regulation of dendritic cell activation and migration from skin explants.. Int J Hyperthermia.

[34] Zheng H, Benjamin IJ, Basu S, Li Z (2003). Heat shock factor 1-independent activation of dendritic cells by heat shock: implication for the uncoupling of heat-mediated immunoregulation from the heat shock response.. Eur J Immunol.

[35] Basu S, Srivastava PK (2003). Fever-like temperature induces maturation of dendritic cells through induction of hsp90.. Int Immunol.

[36] Tournier JN, Hellmann AQ, Lesca G, Jouan A, Drouet E (2003). Fever-like thermal conditions regulate the activation of maturing dendritic cells.. J Leukoc Biol.

[37] DeFillipo AM, Dai J, Li Z (2004). Heat shock-induced dendritic cell maturation is coupled by transient aggregation of ubiquitinated proteins independently of heat shock factor 1 or inducible heat shock protein 70.. Mol Immunol.

[38] Singh IS, Viscardi RM, Kalvakolanu I, Calderwood S, Hasday JD (2000). Inhibition of tumor necrosis factor-alpha transcription in macrophages exposed to febrile range temperature. A possible role for heat shock factor-1 as a negative transcriptional regulator.. J Biol Chem.

[39] Belyakov IM, Hammond SA, Ahlers JD, Glenn GM, Berzofsky JA (2004). Transcutaneous immunization induces mucosal CTLs and protective immunity by migration of primed skin dendritic cells.. J Clin Invest.

[40] Martin MP, Seth S, Koutsonanos DG, Jacob J, Compans RW (2010). Adjuvanted influenza vaccine administered intradermally elicits robust long-term immune responses that confer protection from lethal challenge.. PLoS One.

[41] Campos-Neto A, Porrozzi R, Greeson K, Coler RN, Webb JR (2001). Protection against cutaneous leishmaniasis induced by recombinant antigens in murine and nonhuman primate models of the human disease.. Infect Immun.

