# Supplementary figures and images for: Adjuvant Properties of Thermal Component of Hyperthermia Enhanced Transdermal Immunization: Effect on Dendritic Cells
Source: PLoS One. 2012 Feb 20;7(2):e32067. doi: 10.1371/journal.pone.0032067 (PMC3282786; doi:10.1371/journal.pone.0032067)

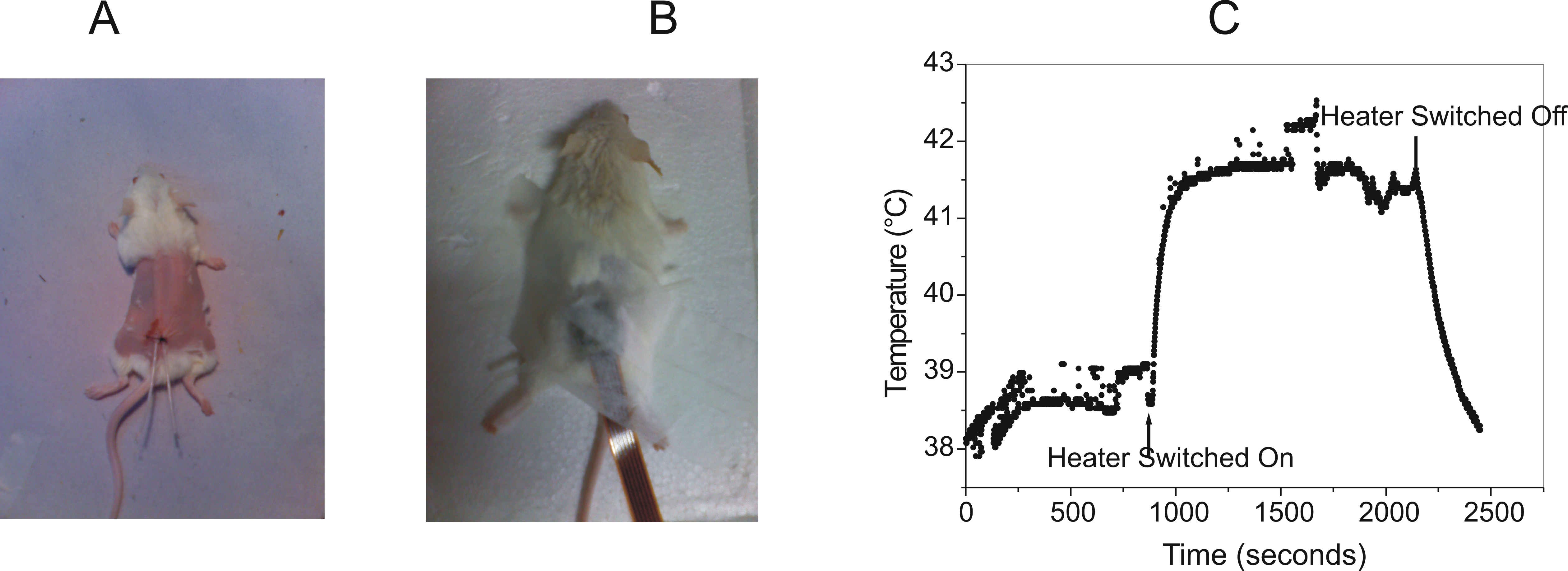

Supplement: Figure S1 — The confirmation of protocol for local hyperthermia. Panel A shows the surgical implantation of the thermistor probe in the mice and panel B depicts how the heating patch to generate hyperthermia was placed over the skin. Panel C shows the temperature profile generated upon treating the mice with local hyperthermia at 42°C which confirms that the temperature beneath skin immediately attains the desired temperature. (TIF) [file pone.0032067.s001.tif]

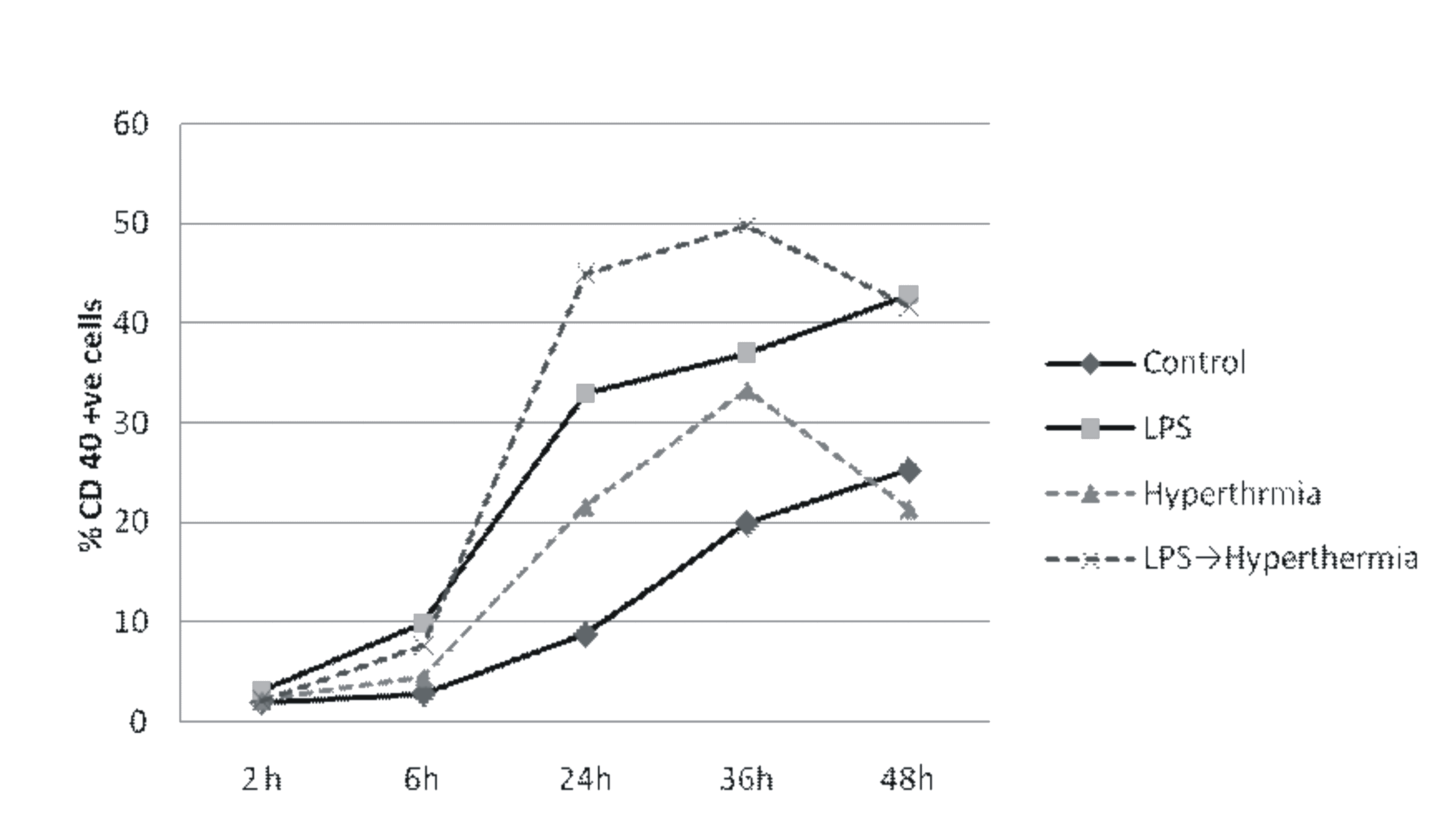

Supplement: Figure S2 — Kinetics of expression of CD40 on BMDCs. Hyperthermia enhances the expression of CD 40 maturation marker on DCs. BMDCs were given in-vitro hyperthermia (42°C, 30 minutes) with or without LPS maturation. In absences of hyperthermia the expression of marker peaks after 48 hours whereas upon hyperthermia stimulation, the peak appears at 36 hours of LPS stimulation. (TIF) [file pone.0032067.s002.tif]
